# The earliest record of Caribbean frogs: a fossil coquí from Puerto Rico

**DOI:** 10.1098/rsbl.2019.0947

**Published:** 2020-04-08

**Authors:** David C. Blackburn, Rachel M. Keeffe, María C. Vallejo-Pareja, Jorge Vélez-Juarbe

**Affiliations:** 1 Florida Museum of Natural History, University of Florida, Gainesville, FL 32611, USA; 2 Department of Biology, University of Florida, Gainesville, FL 32611, USA; 3 Department of Mammalogy, Natural History Museum of Los Angeles County, Los Angeles CA, USA

**Keywords:** Anura, Caribbean, Eleutherodactylidae, fossil

## Abstract

The nearly 200 species of direct-developing frogs in the genus *Eleutherodactylus* (the Caribbean landfrogs, which include the coquís) comprise an important lineage for understanding the evolution and historical biogeography of the Caribbean. Time-calibrated molecular phylogenies provide indirect evidence for the processes that shaped the modern anuran fauna, but there is little direct evidence from the fossil record of Caribbean frogs about their distributions in the past. We report a distal humerus of a frog from the Oligocene (approx. 29 Ma) of Puerto Rico that represents the earliest known fossil frog from any Caribbean island. Based on its prominent rounded distal humeral head, distally projecting entepicondyle, and reduced ectepicondyle, we refer it to the genus *Eleutherodactylus*. This fossil provides additional support for an early arrival of some groups of terrestrial vertebrates to the Greater Antilles and corroborates previous estimates based on molecular phylogenies suggesting that this diverse Caribbean lineage was present in the islands by the mid-Cenozoic.

## 1. Background

Biogeographers have long been interested in how the geological history of the Caribbean has shaped the biota of its islands. Studies combining modern and palaeontological distributions of taxa with an understanding of the complex geological history of the Caribbean have shaped hypotheses explaining the origin of taxa found in the Greater and Lesser Antilles [1,2]. More recently, molecular genetic and phylogenetic studies of small animals have been used to test hypotheses of dispersal overwater or via land-bridges as well as vicariance scenarios [3–8] related to islands at the northern edge of the Caribbean plate as it collided with the North American plate during the Cenozoic [9–11]. Yet the poor fossil record of small terrestrial animals from the early Cenozoic of the Caribbean provides limited direct evidence to corroborate the historical biogeography derived from time-calibrated molecular phylogenetic analyses.

An excellent opportunity for understanding these biogeographic patterns is provided by the rich modern Caribbean frog fauna comprising more than 240 species spread across all of the major island groups [12]. Based on time-calibrated molecular phylogenetic analyses, at least some of the anurans found today on the Hispaniolan and Puerto Rican banks are estimated to have reached there by the Oligocene [13–15]. Approximately two-thirds of all Caribbean frog species are Greater Caribbean landfrogs (genus *Eleutherodactylus*), part of a large clade now called Terraranae [12,16,17]. On the Caribbean islands, these predominantly direct-developing and terrestrial species occupy a range of microhabitats, leading to their characterization as an adaptive radiation [18–20].

Despite the rich extant fauna and its wide distribution, the fossil record provides little insights into the estimated arrival of *Eleutherodactylus* in the Caribbean. Time-calibrated molecular phylogenetic analyses provide contrasting views on the origin of *Eleutherodactylus*, ranging from a recent suggestion of a latest Oligocene or early Miocene colonization of the Caribbean [20] and previous estimates suggesting a mid-Oligocene or even earliest Cenozoic colonization [14]. Our current understanding of the Caribbean fossil record for the genus is limited to the Neogene, consisting of an amber-preserved fossil from the Miocene of the Dominican Republic [21,22], an isolated ilium from the Miocene of Florida [23,24; but see 25], and assorted elements from the Pleistocene–Holocene of Antigua and Barbuda, The Bahamas, Guadeloupe, Jamaica and Puerto Rico [26–31]. Here, we report the oldest anuran record for the Caribbean, which provides direct evidence for the presence of *Eleutherodactylus* in the Greater Antilles by the mid-Cenozoic (approx. 29 Ma).

## 2. Systematic palaeontology

Anura Fischer von Waldheim 1813

Eleutherodactylidae Lutz 1954

*Eleutherodactylus* Duméril and Bibron, 1841

### (a) Specimen

LACM 162445, distal end of left humerus; collected by J. Vélez-Juarbe, 20 November 2012.

### (b) Locality and age

**Figure 1.**
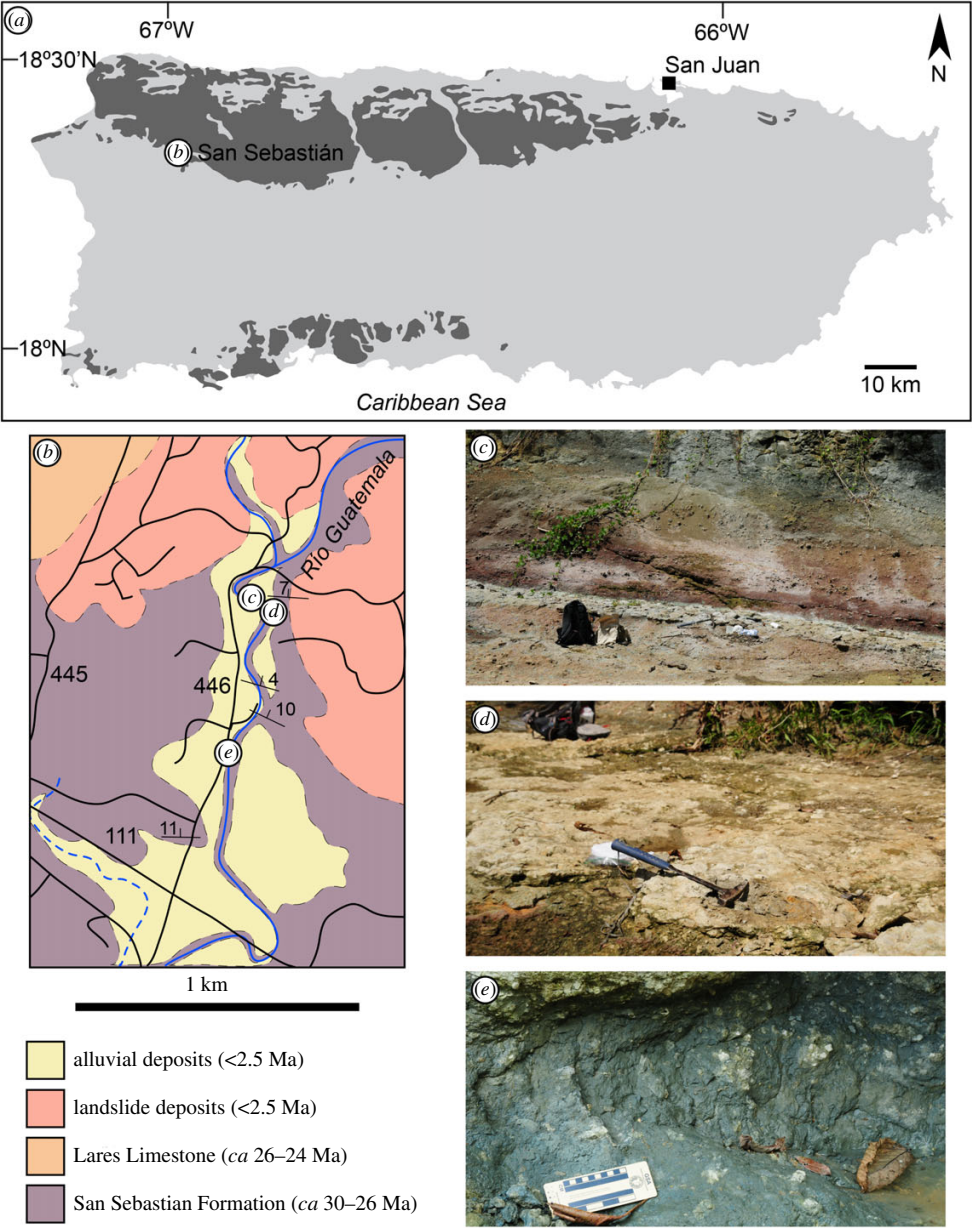
Map of Puerto Rico showing distribution of Oligocene to Pliocene deposits (dark grey) and the location of the town of San Sebastián (*a*) (modified from [32]). (*b*) Geological map of the study area (simplified from [33]), showing relevant exposures of the San Sebastian Formation shown in (*c*–*e*) (solid black lines denote roads). (*c*) Type locality of *Priscosiren atlantica*. Other vertebrates from this level include pelomedusoid turtles and caviomorph rodents [34–36]. (*d*) Fossil invertebrate bed. Strontium dating of *Kuphus incrassatus* tubes found *in situ* in this unit yielded an age of 29.47 ± 0.30 Ma [37]. (*e*) LACM Loc. 8059, mudstone unit of the basal San Sebastian Formation where *Eleutherodactylus* sp. (LACM 162445) was collected.

Specimen was collected from LACM Loc. 8059, a small outcrop on west bank of Rio Guatemala, San Sebastián, Puerto Rico (figure 1); 18°21'02.59″ N, 66°59'48.60″ W. This horizon consists of a bluish grey mudstone of the basal San Sebastian Formation as exposed along Río Guatemala, Puerto Rico. The lithology of this horizon, as well as the over- and underlaying horizons, indicates terrestrial to shallow aquatic habitats within coastal deltaic settings. The San Sebastian Formation along Río Guatemala has yielded a vertebrate fauna that so far includes sharks, fishes, gharials, turtles, sirenians and caviomorph rodents [34–36,38]. The age of LACM 162445 is estimated to be 30.0–29.5 Ma (Early Oligocene). This is based on invertebrate shells from about 80 m stratigraphically above this level that have been strontium-dated as 29.47 ± 0.30 Ma ([37], figure 1).

### (c) Description

**Figure 2.**
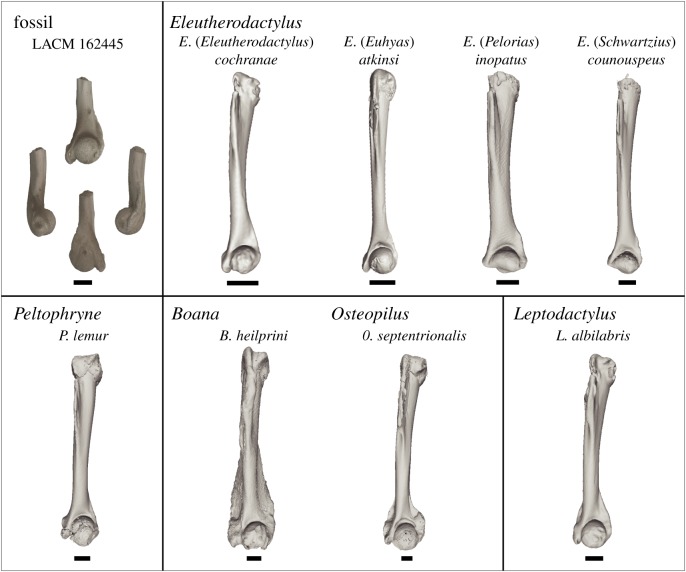
Comparisons of LACM 162445 to representatives of each extant Caribbean frog genus as well as each Caribbean subgenus of *Eleutherodactylus* (*Eleutherodactylus*, *Euhyas*, *Pelorias* and *Schwartzius*). Clockwise from top, LACM 162445 is in anterior, medial, posterior and lateral views. Information on specimens is provided in electronic supplementary material, table S1. Scale bars, 1 mm.

LACM 162445 preserves the distal third of a left humerus (figure 2). There is a large, prominent, and round distal humeral head that is wider (1.34 mm) than the width of the diaphysis (0.83 mm). The entepicondyle (ulnar epicondyle) is well-developed and projects beyond the distal margin of the condyle. The crests along the medial and lateral surfaces of the diaphysis extending towards the ent- and ectepicondyles, respectively, are differently developed with the lateral one being more defined. The ectepicondyle (radial epicondyle) is poorly developed and is represented as a small ridge extending from the diaphysis and along the lateral surface of the condyle.

We compared LACM 162445 with representatives of all extant anuran genera native to the Caribbean [12] (figure 2; electronic supplementary material, table S1), including *Boana* and *Osteopilus* (Hylidae), *Leptodactylus* (Leptodactylidae) and *Peltophryne* (Bufonidae), as well as each of the subgenera of *Eleutherodactylus* and representatives of its species series and species subgroups (electronic supplementary material, figure S1) on the Puerto Rican Bank [16]. Comparisons were made based on data resulting from microcomputed tomography scanning (electronic supplementary material). LACM 162445 differs from all modern Caribbean genera except *Eleutherodactylus* by having an entepicondyle that projects beyond the distal margin of the humeral head. In comparison with the hylids *Boana* and *Osteopilus*, LACM 162445 lacks well-developed lateral and medial crests along the distal diaphysis; the medial crest is also more developed in the leptodactylid *Leptodactylus*. The distally projecting entepticondyle also differentiates LACM 162445 from other eleutherodactylids, incuding *Adelophryne*, *Diasporus* and *Phyzelaphryne* as well as other non-Caribbean terraranans such as *Pristimantis* and *Strabomantis* (electronic supplementary material, figure S2).

Among the modern Caribbean genera examined, LACM 162445 has the clearest similarities to the species-rich genus *Eleutherodactylus* (figure 2). The combination of being a well-ossified humerus with a well-developed and rounded distal humeral head, lack of prominent medial and lateral crests, and a distally projecting entepicondyle, all suggest that LACM 162445 is referable to the genus *Eleutherodactylus*. Both the rounded distal humeral head and an entepicondyle projecting beyond the humeral head differ from that of the subgenera *Pelorias* and *Schwartzius*, both endemic to Hispaniola. LACM 162445 has the clearest similarities to species of three *Eleutherodactylus* subgenera (electronic supplementary material, figure S1), *Eleutherodactylus*, *Euhyas* and *Syrrophus*, which do not form a clade [17]. In several species of these genera, the distal humeral head is rounded and associated with a well-defined entepicondyle projecting beyond its distal margin.

The adult body size of LACM 162445 is relatively smaller than observed in adults of other modern Caribbean genera, all of which attain body sizes of above 45 mm snout–urostyle length (electronic supplementary material, table S1). Based on comparisons with the modern *Eleutherodactylus* sampled here, LACM 162445 likely represents an adult owing to its well-ossified distal humeral head and entepicondyle. For comparison, note the subadult specimen of *E. johnstonei* in electronic supplementary material, figure S1, for which the epicondyles are not synostosed to the diaphysis. Extrapolating from the strong positive relationship between humeral head width and snout–urostyle length among modern *Eleutherodactylus* (electronic supplementary material, figure S3 and table 2), this extinct frog was likely a medium-sized species (approx. 36 mm snout-urostyle length) that falls in the lower half of the size range of extant species of *Eleutherodactylus* (11–88 mm snout–vent length) [16].

This new record of the oldest Caribbean frog fossil provides direct evidence that the genus *Eleutherodactylus*—representing the vast majority of the modern Caribbean frog fauna—was present on the Puerto Rican Bank by the early Oligocene (approx. 29 Ma). Evidence for complete submergence of this bank since the Oligocene is lacking, and it has most likely been subaerially exposed since at least the late Eocene–early Oligocene [39]. This supports an older colonization than suggested by the most recent time-calibrated molecular phylogenetic study [20], but corroborates similar past studies suggesting that *Eleutherodactylus* was established in the Greater Antilles during the Eocene or early Oligocene, with the major lineages recognized as subgenera diversifying approximately 20–40 Ma [3,14]. This fossil may represent crown-group *Eleutherodactylus* or instead a closely related stem taxon. Thus, while it provides direct evidence that this lineage was present in the Greater Antilles by the early Oligocene, it does not directly inform us as to the early diversification of this genus.

In general, there are limited records of small terrestrial vertebrates from the Palaeogene and Neogene of the Greater Antilles. However, with few exceptions [40], Oligocene–Miocene terrestrial vertebrates from Puerto Rico, Hispaniola and Cuba have extant representatives or are closely related to taxa that became extinct during the Pleistocene (electronic supplementary material, table S3). The presence of *Eleutherodactylus* in the early Oligocene of Puerto Rico is consistent with overwater dispersal or, alternatively, the hypothesized presence of a geologically short-lived land connection between northern South America and Puerto Rico, Hispaniola and eastern Cuba (GAARlandia land span hypothesis) at or near the Eocene–Oligocene boundary [2,10]. The presence of this land connection, or alternatively, a set of closely spaced islands, during this time would have facilitated colonization of terrestrial taxa from South America to the Greater Antilles [4,41,42]. Although some of the geological evidence for the presence of the GAARlandia land span is still lacking [43], other palaeontological and molecular evidence is consistent with the synchronous arrival of terrestrial organisms to the region approximately 33.9 ± 1 Ma, while others clearly arrived through multiple overwater dispersal events throughout the Neogene or even earlier [8,13,15,35,36,44–50]. Furthermore, molecular phylogenetic studies have suggested a similar Oligocene arrival for at least some anuran taxa on the Puerto Rican and Hispaniolan banks, such as *Leptodactylus* [13] and *Peltophryne* [15]. The persistence of *Eleutherodactylus* in the Greater Antilles since the Oligocene is evidenced by its presence in the Miocene amber deposits in Hispaniola [21,22].

Finally, a potential argument against the presence of a land span is the low taxonomic diversity during the early Oligocene of the Greater Antilles [4]. However, following the initial discovery of an Oligocene sloth [2], more continuous effort aimed at finding and documenting additional terrestrial and semi-aquatic taxa from this time period have only been conducted over the last 14 years and across a few available localities [35,36,38], including the one documented here. Continuation of this fieldwork in Palaeogene deposits in Puerto Rico and across the Caribbean may reveal other instances of early arrivals and further improve our understanding of the origins of the Greater Antillean terrestrial fauna.

## Supplementary Material

Supplemental Materials

## Supplementary Material

Figure S1

## Supplementary Material

Figure S2

## Supplementary Material

Figure S3

## Data accessibility

CT scan data are available through www.morphosource.org. See the electronic supplementary material, including table S1, for further details and dois.
