# Supplementary material for: The earliest record of Caribbean frogs: a fossil coquí from Puerto Rico
Source: Biol Lett. 2020 Apr 8;16(4):20190947. doi: 10.1098/rsbl.2019.0947 (PMC7211465; doi:10.1098/rsbl.2019.0947)
Supplement: Supplemental Materials [file rsbl20190947supp1.docx]

Electronic Supplementary Material for

**The earliest record of Caribbean frogs: a fossil coquí from Puerto Rico**

DAVID C. BLACKBURN^1,^*, RACHEL M. KEEFFE^1,2^, MARÍA CAMILA VALLEJO-PAREJA^1,2^, and JORGE VÉLEZ-JUARBE^3^

^1^Florida Museum of Natural History, University of Florida, Gainesville, FL 32611, U.S.A.

^2^Department of Biology, University of Florida, Gainesville, FL 32611, U.S.A.

^3^Department of Mammalogy, Natural History Museum of Los Angeles County, 900 Exposition Blvd., Los Angeles, CA 90007, U.S.A.

*Author for correspondence. Email: dblackburn@flmnh.ufl.edu

**Methods**

We selected extant species for comparisons to LACM 162445 based on the presence of distribution of the modern anuran genera in the Caribbean (Hedges et al. 2019). In addition, we made comparisons of the fossil to all subgenera of *Eleutherodactylus*, all relevant species series, species groups, and species subgroups of *Eleutherodactylus* on the Puerto Rican bank (Hedges et al., 2008), representatives of other genera of Eleutherodactylidae (*Adelophryne*, *Diasporus*, and *Phyzelaphryne*), and representatives of the Strabomantidae (*Pristimantis*, *Strabomantis*). Specimens in our study were derived from scientific collections of the California Academy of Sciences (CAS; San Francisco, CA, USA), Field Museum of Natural History (FMNH; Chicago, IL, USA), Florida Museum of Natural History, University of Florida (UF; Gainesville, FL, USA), National Museum of Natural History, Smithsonian Institution (NMNH; Washington, DC, USA), and University of Kansas Natural History Museum (KU; Lawrence, KS, USA).

We performed high-resolution computed tomography scanning of both the fossil specimen and fluid-preserved adult specimens of extant species at the University of Florida’s Nanoscale Research Facility using a Phoenix v|tome|x M (GE Measurement & Control Solutions, Boston, Massachusetts, USA). All scans were run using a 180-kv X-ray tube containing a diamond-tungsten target, with the voltage, current, and detector capture time adjusted for each scan to maximize absorption range for each specimen. Raw X-ray data were processed using GE’s proprietary datosjx software v.2.3 to produce a series of tomogram images and volumes. The resulting microCT volume files were imported into VG StudioMax v.3.0 (Volume Graphics, Heidelberg, Germany), the humeri isolated using VG StudioMax’s suite of segmentation tools, and then exported as high-fidelity shapefiles (.ply format). We deposited image stacks (TIFF) and 3D mesh files (STL) in MorphoSource (Supplementary Table 2).

We took measurements of 24 species of *Eleutherodactylus* in VG StudioMax v.3.0 using the Polyline length measurement tool. Snout–urostyle length (SUL) was taken from the medial tip of the left premaxilla to the posterior-most part of the urostyle. Humerus head width (HHW) was measured on left humeri at the middle of the condyle from lateral to medial side, between the ulnar and radial epicondyles.

We used a regression analysis of SUL and HHW to predict the SUL of the fossil *Eleutherodactylus* from Puerto Rico, we used R (R Core team, 2013). We natural log-transformed the measurements and then calculated the correlation and significance of correlation of the two variables. The correlation coefficient (r) is 0.97 and the Pearson correlation test indicates that the correlation is significant (P-value= 1.85^-14)^. We then performed a linear regression using an ordinary least square regression method (OLS) with HHW as the independent variable. We used the resulting regression formula to estimate the SUL of LACM 162445 based on the width of the humeral head.

**Supplemental Figures**

**Supplemental Figure 1.** Comparisons of LACM 162445 to representative species of the currently recognized subgenera of *Eleutherodactylus* (*Eleutherodactylus*, *Euhyas*, *Pelorias*, *Schwartzius*, and *Syrrophus*). Information on specimens provided in Supplementary Table 1. Scale bars, 1 mm.

**Supplemental Figure 2.** Comparisons of LACM 162445 to representatives of other genera of the Eleutherodactylidae and Strabomantidae (another “terraranan” family). Information on specimens provided in Supplementary Table 1. Scale bars, 1 mm.

**Supplemental Figure 3.** Linear regression (R function) of snout–urostyle length (SUL) on humeral head width (both variables natural log-transformed) for extant representatives of subgenera of *Eleutherodactylus*. The coefficient of determination (*r*^2^) is high 0.934 (adjusted *r*^2^= 0.93; *P* < 0.0001; F_1,22_ = 310.4) of the resulting relationship (lnSUL = 3.31925 + 0.8883971[lnHHW]) allows us to estimate SUL for LACM 162445 as ~36 mm (star plotted at lnSUL = 3.58), based on humeral head width of 1.34 mm.

**Supplementary Tables**

Supplementary Table 2. Measurements (in millimeters) of snout–urostyle length (SUL) and humeral head width (HHW) for representative species of *Eleutherodactylus*.

| Subgenus | Species | Catalog number | Snout–Urostyle (mm) | Humeral Head Width (mm) |
| --- | --- | --- | --- | --- |
| *Eleutherodactylus* | *Eleutherodactylus antillensis* | UF:herp:100862 | 22.02 | 0.86 |
| *Eleutherodactylus* | *Eleutherodactylus cochranae* | UF:herp:153063 | 23.24 | 0.83 |
| *Eleutherodactylus* | *Eleutherodactylus coqui* | UF:herp:21290 | 32.65 | 1.19 |
| *Eleutherodactylus* | *Eleutherodactylus eneidae* | UF:herp:100532 | 25.01 | 0.85 |
| *Eleutherodactylus* | *Eleutherodactylus gryllus* | KU:kuh:279279 | 17 | 0.53 |
| *Eleutherodactylus* | *Eleutherodactylus hedricki* | UF:herp:137271 | 47.26 | 1.73 |
| *Eleutherodactylus* | *Eleutherodactylus jasperi* | KU:kuh:280595 | 18.67 | 0.72 |
| *Eleutherodactylus* | *Eleutherodactylus johnstonei* | UF:herp:27911 | 23.96 | 0.8 |
| *Eleutherodactylus* | *Eleutherodactylus karlschmidti* | UF:herp:24198 | 62.36 | 2.89 |
| *Eleutherodactylus* | *Eleutherodactylus portoricensis* | UF:herp:103249 | 27.75 | 1.15 |
| *Eleutherodactylus* | *Eleutherodactylus richmondi* | UF:herp:100531 | 33.81 | 1.15 |
| *Euhyas* | *Eleutherodactylus atkinsi* | UF:herp:177996 | 30.92 | 0.95 |
| *Euhyas* | *Eleutherodactylus dimidiatus* | UF:herp:21949 | 16.96 | 0.65 |
| *Euhyas* | *Eleutherodactylus eunaster* | CAS:herp:119580 | 30.02 | 1.22 |
| *Euhyas* | *Eleutherodactylus glaphycompus* | UF:herp:56811 | 33.86 | 0.99 |
| *Euhyas* | *Eleutherodactylus lentus* | KU:kuh:94394 | 34.21 | 1.02 |
| *Euhyas* | *Eleutherodactylus monensis* | UF:herp:39548 | 20.01 | 0.73 |
| *Euhyas* | *Eleutherodactylus orientalis* | UF:herp:167747 | 11.94 | 0.42 |
| *Euhyas* | *Eleutherodactylus ricordii* | UF:herp:179256 | 17.85 | 0.65 |
| *Euhyas* | *Eleutherodactylus turquinensis* | USNM:348804 | 22.54 | 0.87 |
| *Euhyas* | *Eleutherodactylus cuneatus* | USNM:5202 | 35.19 | 1.34 |
| *Pelorius* | *Eleutherodactylus inoptatus* | UF:herp:42264 | 37.27 | 1.36 |
| *Schwartzius* | *Eleutherodactylus counouspeus* | UF:herp:100966 | 41.36 | 1.56 |
| *Syrrhophus* | *Eleutherodactylus pipilans* | UF:herp:104284 | 21.92 | 0.77 |

| Supplementary Table 3. Terrestrial vertebrates from the Oligocene-Miocene of the Greater Antilles (modified and updated from [1]:table 1). | | | |
| --- | --- | --- | --- |
| Taxa | Formation/Age | Country | Literature |
| **Anura, Eleutherodactylidae** | | | |
| *Eleutherodactylus* sp. | San Sebastian/early Oligocene (>29.78 Ma) | PR | [2]; This work |
| *Eleutherodactylus* sp. | La Toca Fm./early-middle Miocene (~20–15 Ma) | DR | [3–4] |
| **Anura, Leptodactylidae** | | | |
| Leptodactylidae indet. | Amber mine in Cordillera Septentrional/early-middle Miocene (~20–15 Ma) | DR | [4–5] |
| **Caudata, Plethodontidae** | | | |
| *Palaeoplethodon hispaniolae* | Amber mine in Cordillera Septentrional/early-middle Miocene (~20–15 Ma) | DR | [4,6] |
| **Gekkota, Sphaerodactylidae** | | | |
| *Sphaerodactylus dommeli* | La Toca Fm./early-middle Miocene (~20–15 Ma) | DR | [4,7–8] |
| *Sphaerodactylus ciguapa* | La Toca Fm./early-middle Miocene (~20–15 Ma) | DR | [4,9] |
| *Sphaerodactylus* spp. | Amber mines in Cordillera Septentrional/early-middle Miocene (~20–15 Ma) | DR | [4,10] |
| **Iguania, Dactyloidae** | | | |
| *Anolis dominicanus* | La Toca Fm./early-middle Miocene (~20–15 Ma) | DR | [4,11] |
| *Anolis* sp. cf. *chlorocyanus* sp. group | La Toca Fm./early-middle Miocene (~20–15 Ma) | DR | [4,12] |
| *Anolis* spp. | Amber mines in Cordillera Septentrional/early-middle Miocene (~20–15 Ma) | DR | [4,13–14] |
| **Iguania, Iguanidae** | | | |
| ?Iguanidae indet. | Cibao Fm./early-middle Miocene (~16–12.17 Ma) | PR | [2,15] |
| **Serpentes, Boidae** | | | |
| Boidae indet. | Cibao Fm./early-middle Miocene (~16–12.17 Ma) | PR | [2,15] |
| **Eulipotyphla, Solenodonota** | | | |
| Solenodonota gen. et sp. indet. | La Toca Fm./early-middle Miocene (~20–15 Ma) | DR | [4,16] |
| **Xenarthra, Folivora** | | | |
| ?Folivora indet. | Juana Diaz Fm./early Oligocene (30.3-27.5 Ma) | PR | [17–18] |
| *Imagocnus zazae* | Lagunitas Fm./early Miocene (~18.5–17.5 Ma) | CU | [1,19] |
| **Primates, Platyrrhini** | | | |
| *Paralouatta marianae* | Lagunitas Fm./early Miocene (~18.5–17.5 Ma) | CU | [1,17] |
| **Rodentia, Caviomorpha** | | | |
| *Borikenomys praecursor* | San Sebastian/early Oligocene (29.78–26.51 Ma) | PR | [2,20–21] |
| Chinchilloid gen. et sp. indet. | San Sebastian/early Oligocene (29.78–26.51 Ma) | PR | [2,21] |
| Caviomorpha gen. et sp. indet. B | Lares Limestone/late Oligocene (26.51–24.73 Ma) | PR | [2,20] |
| **Caviomorpha, Echimyidae, Capromyinae** | | | |
| *Zazamys veronicae* | Lagunitas Fm./early Miocene (~18.5–17.5 Ma) | CU | [1,17] |
| **Aves, Picidae** | | | |
| cf. *Nesoctites* sp. | La Toca Fm./early-middle Miocene (~20–15 Ma) | DR | [4,22] |
| Abbreviations: CU, Cuba; DR, Dominican Republic; PR, Puerto Rico. | | | |

**Supplementary References**

1. MacPhee RDE, Iturralde-Vinent MA, Gaffney ES. 2003 Domo de Zaza, an early Miocene vertebrate locality in south-central Cuba, with notes on the tectonic evolution of Puerto Rico and the Mona Passage. *Am. Mus. Novit.* **3394**. 1–42.
2. Ortega-Ariza D, Franseen EK, Santos-Mercado H, Ramírez-Martínez WR, Core-Suárez EE. 2015 Strontium isotope stratigraphy for Oligocene-Miocene carbonate systems in Puerto Rico and the Dominican Republic: implications for Caribbean processes affecting depositional history. *J. Geol.* **123**, 539–560.
3. Poinar GO, Jr, Cannatella DC. 1987 An Upper Eocene frog from the Dominican Republic and its implication for Caribbean biogeography. *Science* **237**, 1215–1216.
4. Iturralde-Vinent MA, MacPhee RDE. 1996 Age and paleogeographic origin of Dominican amber. *Science* **273**, 1850–1852.
5. Anderson SR. 2004 Insect meals from a leptodactylid frog (Amphibia: Leptodactylidae) in Dominican Amber (Miocene, 23 Ma). *Entomol. News* **115**, 55–57.
6. Poinar G, Jr, Wake DB. 2015 *Palaeoplethodon hispaniolae* gen. n., sp. n. (Amphibia: Caudata), a fossil salamander from the Caribbean. *Palaeodiversity* **8**, 21–29.
7. Böhme W. 1984. Erstfund eines fossilien Kugelfingergeckos (Sauria: Gekkonidae: Sphaerodactylinae) aus Dominikanischem Bernstein (Oligozän von Hispaniola, Antillen). *Salamandra* **20**, 212–220.
8. Daza JD, Bauer AM, Snively ED. 2014 On the Fossil record of the Gekkota. *Anat. Rec.* **297**, 433–462.
9. Daza JD, Bauer AM. 2012 A new amber-embedded sphaerodactyl gecko from Hispaniola, with comments on morphological synapomorphies of the Sphaerodactylidae. *Breviora* **529**, 1–28.
10. Daza JD, Bauer AM, Snively ED. 2014 On the Fossil record of the Gekkota. *Anat. Rec.* **297**, 433–462.
11. Rieppel O. 1980 Green anole in Dominican amber. *Nature* **286**,486–487.
12. de Queiroz K, Chu LR, Losos JB. 1998 A second *Anolis* lizard in Dominican amber and the systematics and ecological morphology of Dominican amber anoles. *Am. Mus. Novit.* **3249**, 1–23.
13. Polcyn MJ, Rogers JV, II, Kobayashi Y, Jacobs LL. 2002 Computed tomography of an *Anolis* lizard in Dominican amber: systematic, taphonomic, biogeographic, and evolutionary implication. *Paleontol. Electron.* **5**, 1–13.
14. Sherratt E, Castañeda M del R, Garwood RJ, Mahler DL, Sanger TJ, Herrel A, de Queiroz K, Losos JB. 2015 Amber fossils demonstrate deep-time stability of Caribbean lizard communities. *P. Natl. Acad. Sci. USA* **112**, 9961–9966.
15. MacPhee RDE, Wyss AR. 1990 Oligo-Miocene vertebrates from Puerto Rico, with a catalog of localities. *Am. Mus. Novit.* **2965**, 1–45.
16. MacPhee RDE, Grimaldi DA. 1996 Mammal bones in Dominican amber. *Nature* **380**, 489–490.
17. MacPhee RDE, Iturralde-Vinent MA. 1995 Origin of the Greater Antillean land mammal fauna, 1: new Tertiary fossils from Cuba and Puerto Rico. *Am. Mus. Novit.* **3141**, 1–31.
18. Schweitzer CE, Iturralde-Vinent M, Hetler JL, Velez-Juarbe J. 2006 Oligocene and Miocene decapods (Thalassinidea and Brachyura) from the Caribbean. *Ann. Carnegie Mus.* **75**, 111–136.
19. MacPhee RDE, Iturralde-Vinent MA. 1994 First Tertiary land mammal from Greater Antilles: an early Miocene sloth (Xenarthra, Megalonychidae) from Cuba. *Am. Mus. Novit.* **3094**, 1–13.
20. Vélez-Juarbe J, Martin T, MacPhee RDE, Ortega-Ariza D. 2014 The earliest Caribbean rodents: Oligocene caviomorphs from Puerto Rico. *J. Vertebr. Paleontol.* **34**, 157–163.
21. Marivaux L, Velez-Juarbe J, Merzeraud G, Pujos F, Viñola López LW, Boivin M, Santos-Mercado H, Cruz EJ, Grajales A, Padilla J, Vélez-Rosado KI, Philippon M, Léticée J-L, Münch P, Antoine P-O. 2020. Early Oligocene chinchilloid caviomorphs from Puerto Rico and the initial rodent colonization of the West Indies. *Proc. R. Soc. B*. **287**, 20192806.
22. Laybourne RC, Deedrick DW, Hueber FM. 1994 Feather in amber is earliest New World fossil of Picidae. *Wilson Bull.* **106**, 18–25.
