# Supplementary figures and images for: The earliest record of Caribbean frogs: a fossil coquí from Puerto Rico
Source: Biol Lett. 2020 Apr 8;16(4):20190947. doi: 10.1098/rsbl.2019.0947 (PMC7211465; doi:10.1098/rsbl.2019.0947)

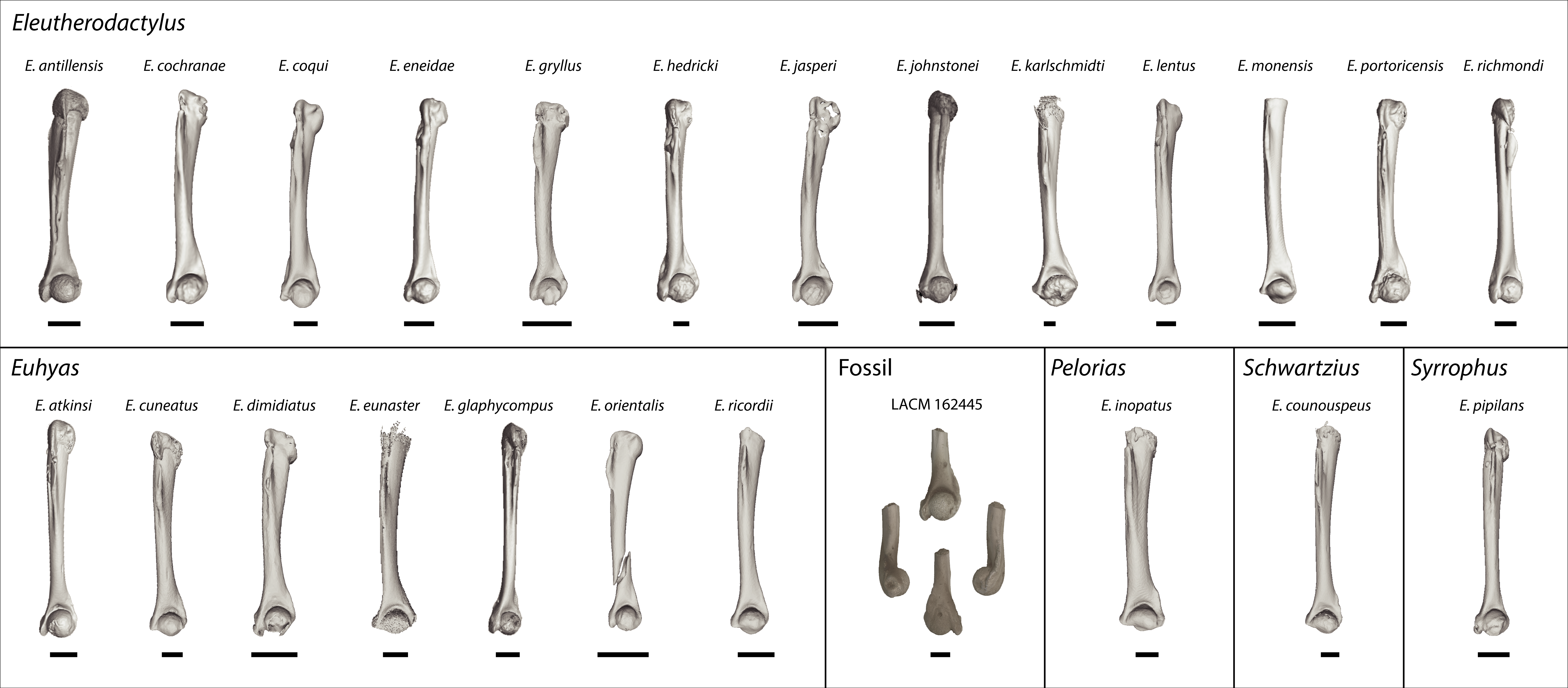

Supplement: Figure S1 [file rsbl20190947supp2.png]

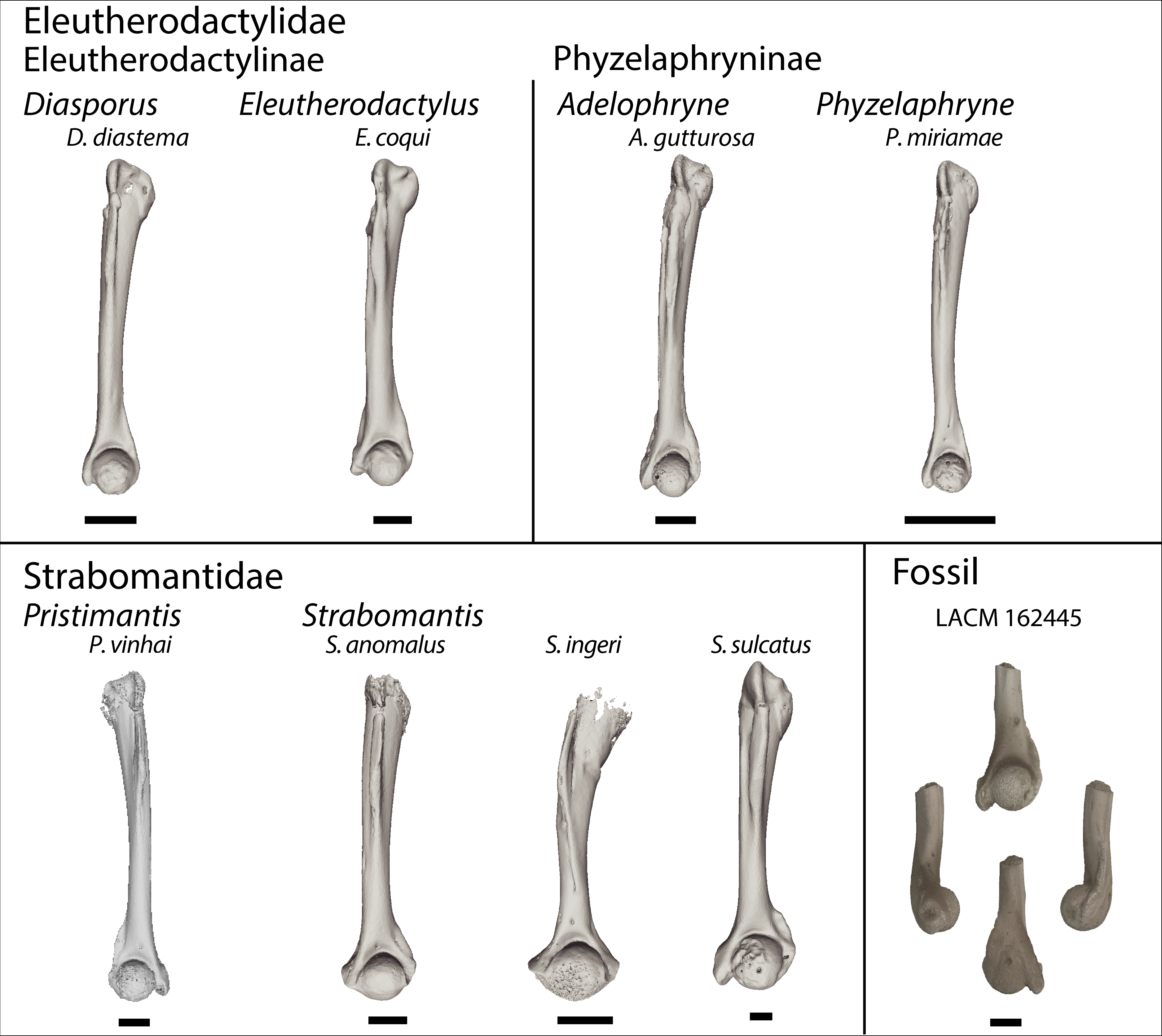

Supplement: Figure S2 [file rsbl20190947supp3.png]

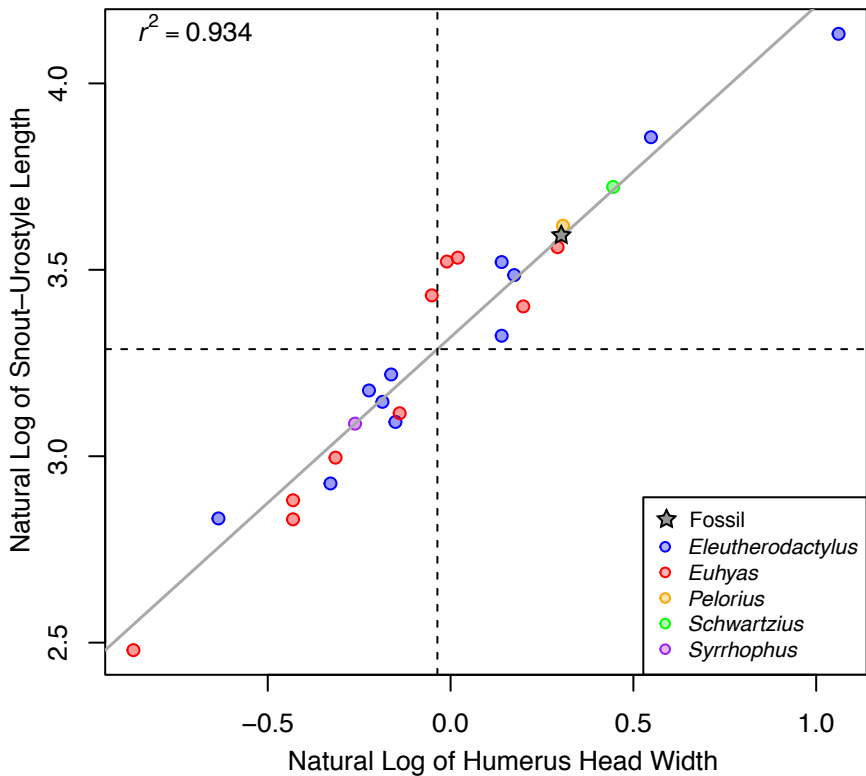

Supplement: Figure S3 [file rsbl20190947supp4.pdf]
